# Impact of the COVID-19 pandemic on the incidence of acute mastoiditis in a tertiary reference children’s hospital in Brazil

**DOI:** 10.1016/j.jped.2025.05.003

**Published:** 2025-06-28

**Authors:** Hemily Izabel Alves Neves, Joel Lavinsky, Luana Vieira Margheti, Mauricio Schneider Miura, Patrícia Barcelos Ogando, Marcelo Assis Moro da Rocha Filho, Jose Faibes Lubianca Neto

**Affiliations:** a Santa Casa de Misericórdia de Porto Alegre, Serviço de Otorrinolaringologia, Porto Alegre, RS, Brazil; b Hospital da Criança Santo Antônio, Serviço de Otorrinolaringologia Pediátrica, Porto Alegre, RS, Brazil

**Keywords:** Mastoiditis, Acute otitis media, Covid-19, Otology, Pediatric otolaryngology

## Abstract

**Objectives:**

To compare the incidence of AM hospitalizations and complications across three periods: pre-pandemic, pandemic, and post-pandemic. Additionally, the study aims to evaluate associations with patient age, need for surgical intervention, antibiotic therapy, and isolated etiological agents.

**Methods:**

This retrospective cohort study reviewed pediatric charts from three 22-month periods: pre-pandemic (P1), pandemic (P2), and post-pandemic (P3). These periods were compared in terms of case numbers, presence and severity of AM complications, patient demographics (age and sex), and treatment approaches.

**Results:**

A total of 9 AM cases were recorded in (P1), 5 in the (P2), and 25 in (P3). This represents a 25.5 % reduction in AM incidence during the pandemic compared to the pre-pandemic period, though this was not statistically significant (*p* = 0.8027). However, a significant 103.3 % increase in AM incidence was noted between the pre- and post-pandemic periods (*p* = 0.0322). No significant differences were found among periods regarding age, sex, complications, case severity, surgical intervention, antibiotic duration, or length of hospitalization.

**Conclusion:**

Although AM incidence slightly declined during the pandemic, the post-pandemic period showed a significant rise in the incidence of cases compared to pre-pandemic values.

## Introduction

On March 11, 2020, the World Health Organization (WHO) declared COVID-19 (Coronavirus Disease 2019) a pandemic, urging global leaders to implement control measures such as mask-wearing, hand hygiene, and social distancing. In response, Brazil and other countries temporarily closed daycare centers and schools, particularly in the initial year of the pandemic. In-person activities gradually resumed throughout 2021 and 2022. Countries that adopted similar measures reported a decrease in respiratory tract infections caused by agents with transmission routes similar to SARS-CoV-2, such as respiratory syncytial virus (RSV), *Streptococcus pneumoniae*, and *Haemophilus influenzae.*^1^

Acute otitis media (AOM) is a common pediatric infection and a leading cause of emergency department visits and antibiotic prescriptions for children in developed countries. By the age of 3, approximately 83 % of children have experienced at least one episode of AOM.^2^

Viral upper respiratory tract infections (URTIs) often precede AOM, causing inflammation of the nasopharynx and auditory tube, increasing bacterial colonization, and impairing mucosal defense mechanisms. Dysfunction of the auditory tube facilitates the entry of pathogens into the middle ear, leading to AOM. Children are particularly predisposed to AOM due to the anatomical characteristics of their auditory tubes, which are more horizontally oriented than those of adults, facilitating the ascent of infectious agents via the nasopharynx.^3^

Acute mastoiditis (AM) is the most common complication of AOM, occurring in approximately 1 in 400 cases (0.24 %).^4^ AM is a suppurative infection of the mastoid air cells, resulting from osteitis-induced bone destruction secondary to the underlying infection. Factors influencing susceptibility include anatomical variations in the temporal bone, age, bacterial flora, and immune status. Pathogenesis involves obstruction of the aditus to the mastoid antrum due to edema or granulation tissue resulting from the acute inflammatory process, preventing adequate drainage of purulent material. The infection spreads through the mastoid periosteum, invading adjacent structures including soft tissue, blood vessels, and nerves, leading to severe and potentially life-threatening complications.^3^^,^^4^

Similar to AOM, children are at higher risk of developing AM due to immature immunity and age-related anatomical differences. In pediatric patients, the mastoid is more pneumatized, with thinner bone trabeculae and a smaller aditus, increasing the predisposition to the accumulation of secretions in the antrum.^4^

Multiple studies have documented a marked global decline in AOM incidence and its complications among children during COVID-19-related social isolation^5^, ^6^, ^7^ At Hospital da Criança Santo Antônio (HCSA), a tertiary pediatric hospital serving Porto Alegre, Rio Grande do Sul, Brazil, fluctuations in AM incidence have been observed over the past five years. A marked decrease occurred during the social distancing period (2020–2021), followed by a resurgence in the past two years. This study aims to analyze trends in AM incidence over recent years, with a particular focus on the impact of the COVID-19 pandemic on pediatric cases.

## Materials and methods

### Study design

This was a retrospective, single-center cohort study conducted at HCSA. The period of analysis (May 2018–October 2023) was subdivided into three phases (pre-pandemic, pandemic, and post-pandemic), each lasting 22 months:

Pre-pandemic period (P1): May 2018 to February 2020, ending with the implementation of social distancing measures at the onset of the pandemic. Pandemic period (P2): March 2020 to December 2021, immediately following the implementation of social isolation measures and ending close to the time after which these measures were progressively relaxed. Post-pandemic period (P3): January 2022 to October 2023.

### Participants

The inclusion criteria considered were hospitalization at HCSA for AM between May 2018 and February 2020, age between 0 and 14 years, clinical and radiological findings consistent with AM, and in-person evaluation by the Pediatric Otolaryngology team at HCSA.

The exclusion criteria included children over 14 years old, diagnosis of AOM without clinical criteria for AM, AM secondary to chronic otitis media, CT findings suggestive of AM (including mastoid air cell opacification on computed tomography or similar descriptions) without clinical criteria, and those with medical records consistent with AM but without in-person evaluation by the Pediatric Otolaryngology team during hospitalization. Both sexes were considered, with no stratification regarding the presence of comorbidities, socioeconomic status, or race/ethnicity.

Both sexes were considered, and there was no stratification regarding the presence of comorbidities, socioeconomic status, or race/ethnicity.

For this study, AM was defined as a case of AOM associated with one or more of the following findings on physical examination: outward protrusion of the auricle with obliteration of the postauricular skin crease; cardinal signs of inflammation (redness, warmth, swelling) in the postauricular region; and/or presence of a retro auricular abscess.^3^

### Data collection

The dataset was sourced from the HCSA electronic medical record system (Tasy EMR®). The *HOW.ai* tool (2022, HealthPlus / GROW, Porto Alegre, RS, Brazil) was used to select medical records of children aged 0 to 14 years who were treated at HCSA from May 1, 2018, through October 31, 2023. The EMR was screened by searching for the mandatory keyword “mastoidite” [mastoiditis] and the optional keywords “otorrinolaringologia” [otolaryngology], “otorrino” [ENT] and “otomastoidite” [otomastoiditis], all in Brazilian Portuguese (the language of the records). These criteria identified 331 patient records. Each record thus obtained was individually screened for eligibility by two of the authors (H.I.A.N. and L.V.M.), who then identified the variables of interest in eligible records. A total of 39 medical records were ultimately selected. The extracted data included age (in months), sex, date of admission, total length of stay, presence of complications, type of complications, need for surgical treatment, type of surgical treatment (when needed), antibiotic therapy, total duration of antibiotic therapy, isolated microbial pathogens, and bacterial susceptibility to major antibiotics.

### Statistical analysis

Qualitative variables were expressed as absolute frequencies, while quantitative variables were presented as means, medians, and interquartile ranges (IQR). The Shapiro-Wilk test was used to assess data distribution normality. Chi-square tests with adjusted standardized residuals or Fisher's exact test were employed to test for associations.

Quantitative variables, as well as incidence and monthly mean of the 3 periods, were compared using the Kruskal-Wallis test (for comparisons between the 3 periods), and the Mann-Whitney U test (for comparison between P1/P2 and P1/P3). The significance level was set at 5 % (*p* = 0.05), and all analyses were performed using IBM SPSS Statistics for Windows, Version 25.0 (Armonk, NY: IBM Corp.).

### Ethical considerations

The study was submitted to the Santa Casa de Misericórdia de Porto Alegre Research Ethics Committee, which waived the informed consent requirement, considering the observational, retrospective design and that all data would be obtained through a review of electronic medical records, without any additional contact with the patients involved. All personal and medical information collected during the study remained confidential and coded, available only to the investigators involved in the study. All reasonable efforts were made to maintain confidentiality regarding the identity of the individual patients.

## Results

Through the screening process, 331 medical records containing the word “mastoidite” were pre-selected. Among these, only 40 presented clinical descriptions compatible with the disease. Of this group, one case was excluded due to a lack of in-person evaluation by the Otolaryngology Team, resulting in 39 eligible cases. The remaining 291 records (88 %) referred to children with tomographic findings suggestive of acute mastoiditis (AM), but lacking corresponding clinical criteria for diagnosis.

One child had multiple hospitalizations for AM, with four distinct episodes in different years across various periods, each requiring admission and surgical intervention.

Of the 39 cases that met the inclusion criteria, 9 (23 %) occurred in the pre-pandemic period (Period 1, P1), 5 (12.8 %) during the pandemic (Period 2, P2), and 25 (64 %) in the post-pandemic period (Period 3, P3). These correspond to average monthly rates of 0.41 cases/month in P1 (95 % CI: 0.0083–0.8099), 0.22 in P2 (95 % CI: 0.0065–0.4481), and 1.13 in P3 (95 % CI: 0.5554–1.7173). The difference in these rates across the three periods was statistically significant (Kruskal–Wallis test, *p* = 0.0042) (Table 1b).

**Table 1:** Incidence and monthly average of acute mastoiditis cases across the study periods - (Statistical significance was set at p < 0.05. Bold values indicate results meeting this threshold.)

a) Incidence of acute mastoiditis per 100,000 hospitalizations in children aged 0–14 years. Kruskal–Wallis test across periods; Mann–Whitney tests for pairwise comparisons (P1 vs. P2 and P1 vs. P3).									
	Cases	Hospitalizations	Incidence (per 100,000 inhab.)	SD	SE	95 % CI	Kruskal–Wallis *p*-value	Relative to P1	Mann-Whitney *p*-value
P1	9	13,435	67	7.139	1.522	23.23ー110.74	**0.0137**	–	
P2	5	10,022	49.90	5.273	1.124	6.17ー93.61		↓ 25.5 %	0.8027
P3	25	18,351	136.2	7.576	1.615	82.86ー189.59		↑ 103.3 %	**0.0322**
Total	39	41,808	93.2						

b) Monthly average of AM cases per 100,000 hospitalizations in children aged 0–14 years. Kruskal–Wallis test across periods; Mann–Whitney tests for pairwise comparisons (P1 vs. P2 and P1 vs. P3).									
	Cases	Hospitalizations	Monthly average	SD	SE	95 % CI	Kruskal–Wallis p-value	Relative to P1	Mann-Whitney p-value
P1	9	13,435	0.41	0.9591	0.2045	0.0083ー0.8099	**0.0042**	–	
P2	5	10,022	0.22	0.5284	0.1127	0.0065ー0.4481		↓ 46.34 %	0.6764
P3	25	18,351	1.13	1.3903	0.2964	0.5554ー1.7173		↑ 175.61 %	**0.0146**
Total	39	41,808	0.84						

During the pandemic, overall hospitalizations and emergency room visits declined, including pediatric admissions. To account for this reduction, the authors analyzed AM incidence relative to the total number of pediatric admissions to the emergency department, ensuring more accurate comparisons between periods.

In Period 1 (P1), AM incidence was 67 per 100,000 inhabitants (95 % CI: 23.23–110.74); in Period 2 (P2), it was 50 per 100,000 inhabitants (95 % CI: 6.17–93.61); and in Period 3 (P3), it was 136 per 100,000 inhabitants (95 % CI: 82.86–189.59). The difference in incidence across the three periods was statistically significant (Kruskal–Wallis test, *P* = 0.0137) (Table 1a).

Between Periods 1 and 2, AM incidence decreased by 25.5 % (67 vs. 50 per 100,000 inhabitants), though this change was not statistically significant (*p* = 0.8027). However, AM incidence increased significantly by 103.3 % between Periods 1 and 3 (67 vs. 136.2 per 100,000 inhabitants) (*p* = 0.0322) (Table 1a). This trend was further confirmed when comparing monthly averages across the periods: a 46.3 % decrease from P1 to P2 (*p* = 0.6764) and a 175.6 % increase from P1 to P3 (*p* = 0.0146) (Table 1b). A visual representation of this data is provided in Supplementary Figure 1.

### Age

The mean patient age was 40 months (SD ±22.4), with a median of 38 months (Table 2). Patient ages ranged from 8 to 85 months (7 years and 1 month), with 4 patients aged ≤ 12 months, 8 aged >12–24 months, 20 aged >24–60 months, and 7 aged >60 months (Table 3). The Kruskal–Wallis test indicated no significant differences among the three groups (*p* = 0.5822, Table 2). Similarly, no significant differences were found in pairwise comparisons: P1 vs. P2 (*p* = 0.6993) and P1 vs. P3 (*p* = 0.4940) using the Mann-Whitney test (Supplementary Data 1).

**Table 2:** Quantitative comparison of patient age, total length of stay, and total duration of antibiotic therapy in the pre-pandemic, pandemic, and post-pandemic periods by median and mean (Kruskal–Wallis test) - (Statistical significance was set at p < 0.05).

	Median [IQR]				Media				
	Overall	P1	P2	P3	Overall	P1	P2	P3	*p*-value
Age (Months)	38 [20; 55]	33 [23; 40]	19 [14; 44]	41 [27; 55]	40 ± 22,4	36.4 ± 19.5	34.6 ± 31	42.4 ± 22.1	0.582
Total duration of antibiotic therapy (days)	25 [20; 35]	23 [22; 42]	20 [19; 21]	28 [20; 35]	12.5 ± 9.6	11.2 ± 8.3	9.8 ± 5.6	13.5 ± 10.7	0.202
Total length of hospital stay (days)	9 [7; 15]	7 [6; 14]	8 [8; 10]	9 [8; 15]	27.5 ± 9.9	29 ± 12.4	20.6 ± 4	28.4 ± 9.5	0.587

**Table 3:** Comparison of qualitative variables in the pre-pandemic, pandemic, and post-pandemic periods (Chi-square test) - (Statistical significance was set at p < 0.05).

		Overall		P1		P2		P3		
		*n*	%	*n*	%	*n*	%	*n*	%	*p*-value
Age range	≤12 months	4	10.3 %	1	11.1 %	1	20.0 %	2	8.0 %	0.786
	13–24 months	8	20.5 %	2	22.2 %	2	40.0 %	4	16.0 %	
	25–60 months	20	51.3 %	5	55.6 %	1	20.0 %	14	56.0 %	
	>60 months	7	17.9 %	1	11.1 %	1	20.0 %	5	20.0 %	
Males		21	53.8 %	6	66.7 %	3	60.0 %	12	48.0 %	0.602
Home antibiotics (any)		30	76.9 %	8	88.9 %	3	60.0 %	19	76.0 %	0.462
Complications	Any	32	82.1 %	8	88.9 %	3	60.0 %	21	84.0 %	0.368
	Epidural abscess	4	10.3 %	2	22.2 %	0	0.0 %	2	8.0 %	0.348
	Subperiosteal abscess	29	74.4 %	8	88.9 %	3	60.0 %	18	72.0 %	0.447
	Bezold's abscess	2	5.1 %	0	0.0 %	1	20.0 %	1	4.0 %	0.244
	Central venous thrombosis	4	10.3 %	0	0.0 %	0	0.0 %	4	16.0 %	0.287
	Cranial nerve palsy	2	5.1 %	0	0.0 %	0	0.0 %	2	8.0 %	0.554
	CNS complications	8	20.5 %	2	22.2 %	0	0.0 %	6	24.0 %	0.474
	Major complications	9	23.1 %	2	22.2 %	1	20.0 %	6	24.0 %	0.979
Surgical intervention	Any surgery	34	87.2 %	9	100 %	4	80.0 %	21	84.0 %	0.411
	Tube tympanostomy	29	74.4 %	7	77.8 %	4	80.0 %	18	72.0 %	0.778
	Mastoidectomy	11	28.2 %	3	33.3 %	1	20.0 %	7	28.0 %	0.900
	Subperiosteal abscess drainage	19	48.7 %	4	44.4 %	2	40.0 %	13	52.0 %	0.850
	Epidural abscess drainage	1	2.6 %	1	11.1 %	0	0.0 %	0	0.0 %	0.181

Age was not significantly associated with the presence or absence of minor or major complications (*p* = 0.226, Table 4).

**Table 4:** Presence of complications according to variables (chi-square and Fisher’s exact tests) - (Statistical significance was set at p < 0.05).

		Total	Complications		*p*-value	CNS complications		*p*-value	Major complications		
		*n*	*n*	%		*n*	%		*n*	%	*p*-value
Age range	≤12 months	4	2	50.0 %	0.226	0	0.0 %	0.214	0	0.0 %	0.128
	13–24 months	8	7	87.5 %		0	0.0 %		0	0.0 %	
	25–60 months	20	18	90.0 %		6	30.0 %		6	30.0 %	
	> 60 months	7	5	71.4 %		2	28.6 %		3	42.9 %	
Sex	M	21	18	85.7 %	0.682	5	23.8 %	0.702	6	28.6 %	0.464
	F	18	14	77.8 %		3	16.7 %		3	16.7 %	
Home antibiotics	Y	30	24	80.0 %	1.000	6	20.0 %	1.000	6	20.0 %	0.406
	N	9	8	88.9 %		2	22.2 %		3	33.3 %	

### Sex

There was a slight male predominance in the overall sample (53.8%), with no significant differences in incidence between sexes across the study periods (*p* = 0.602, Table 3). Similarly, no association was found between sex and complication development (*p* = 0.682, Table 4).

### Complications

Of the 39 included cases, 32 (82 %) developed extra- and/or intracranial complications, including subperiosteal abscess (29 cases), epidural abscess (4 cases), central venous thrombosis (4 cases), Bezold's abscess (2 cases), and cranial nerve palsies (one case each of facial and abducens nerve palsy). Complications were identified in 88.9 % of P1 patients (8 cases), 60 % of P2 patients (3 cases), and 84 % of P3 patients (21 cases), with no statistically significant differences in any complication development across periods (*p* = 0.368) or in major complications (*p* = 0.979). For this study, “major complications” were defined as Bezold’s abscess, epidural abscess, cranial nerve palsy, and central venous thrombosis (Table 3).

There was no correlation between complication development and factors such as sex, age, home antibiotic therapy before hospitalization, or the administration of any specific antibiotic (Table 4).

### Treatment

Most patients (76.9 %) had received home antibiotics before admission. The proportion of patients who received antibiotics prior to hospitalization versus those who did not was similar across periods (*p* = 0.462, Table 3). There was no association between prior antibiotic use and complication development (*p* = 1, Table 4).

The median length of hospital stay was 9 days (range, 4–42 days) (Table 2). All patients required parenteral antibiotics at some point during hospitalization. The median total duration of antibiotic therapy (including home, inpatient, and post-discharge use) was 25 days (range, 14–51 days). No significant differences were observed in hospital stay duration (*p* = 0.587) or total antibiotic therapy duration (*p* = 0.202) across periods (Table 2).

Overall, 87.2 % of patients underwent some form of surgical intervention, with no significant differences among periods (*p* = 0.411). The most common procedure was tympanostomy tube placement (29 children, 74.4 %), followed by subperiosteal abscess drainage (19 children, 48.7 %). Some patients underwent more than one type of surgical procedure (Table 3).

Among the entire sample, 13 patients (33.3 %) were managed conservatively, receiving only tympanostomy tubes or no surgical intervention. Of these patients, 6 had some type of complication and 7 had no complications (Table 3). A conservative approach was deemed appropriate due to a favorable clinical course, with physical examination findings indicating significant improvement, even in patients with complications.

Notably, no patient required surgical reintervention during the same hospitalization period, suggesting a favorable short-term surgical outcome.

### Microbiology

Microbiological samples were obtained from 24 cases, with bacterial growth observed in 12 (50 %). Among these, Streptococcus pneumoniae was identified in 11 cases (91.6 %), and Haemophilus influenzae in one. All isolates were susceptible to vancomycin and resistant to sulfamethoxazole/trimethoprim. Penicillin susceptibility was tested in 9 of the 12 positive cultures, revealing 3 isolates with intermediate sensitivity and 3 with confirmed resistance. Interestingly, all nine cases tested for penicillin susceptibility developed complications, regardless of resistance status. However, due to the limited sample size, no definitive conclusions can be drawn regarding the association between antimicrobial resistance and clinical outcomes.

## Discussion

### Relationship between respiratory infections and incidence of otitis media and acute mastoiditis

AM is a relatively common complication of AOM, affecting 1 in every 400 cases, and requires special attention due to its potential for serious adverse outcomes^4^ Both AOM and AM are associated with acute upper respiratory tract infections and several studies have demonstrated a correlation between the seasonality of respiratory viruses and the incidence of AOM and AM.

Other respiratory viruses that share transmission pathways with SARS-CoV-2 saw a decline in incidence during the pandemic, a time when preventive measures such as mask use and social distancing were implemented to contain the spread of COVID-19.

A single-center retrospective observational study conducted in Germany recorded a significant reduction in the incidence of RSV and Influenza infections in patients admitted and tested in an emergency unit, during the COVID-19 pandemic, between 2020 and 2021^8^ Similar results were observed in Qatar in a single-center retrospective cohort that evaluated data from 2019 to 2023. A reduction in hospitalizations due to lower respiratory tract viral infections was identified during the pandemic, followed by a post-pandemic resurgence.^9^

Unfortunately, the absence of viral serology data (including SARS-CoV-2 co-infections) prevented an evaluation of this pattern in the institution.

### Pneumococcal vaccine and its impact on the incidence of acute otitis and mastoiditis

The present study corroborates findings from similar studies,^10^ identifying *Streptococcus pneumoniae* as the predominant pathogen, isolated in 91.6 % (11/12) of cultures. Due to the retrospective nature of the study, the authors lacked access to vaccination status data, limiting the ability to infer the impact of pneumococcal immunization on the present sample.

However, epidemiological data from Brazil’s Unified Health System (Sistema Único de Saúde - SUS) indicate widespread pediatric immunization with the pneumococcal conjugate vaccine 10 (PCV-10), which has been available free of charge since 2010 for children aged ≥2 months as part of the Brazilian National Vaccination Program (NVP). Data from 2024 show a national vaccination coverage of 92.92 %, reaching 93.92 % in southern Brazil.^11^^,^^12^

A study conducted in Goiânia, Brazil, assessed the impact of PCV10 introduction into the national immunization schedule on AOM incidence in children aged 2–23 months (2008–2015). The results estimated a 43.0 % reduction (95 % CI, 41.4–44.5) in AOM incidence post-PCV10 implementation.^13^

### The COVID-19 pandemic and its impact on the incidence of acute mastoiditis

Several studies have reported a decline in AOM and AM incidence during the pandemic. A cohort study in a tertiary hospital in The Netherlands identified significant reductions in AOM (63 %) and chronic otitis media with effusion (57 %) during social isolation.^5^ Similar results were observed in the UK pediatric population^6^ and in Shanghai, where a 63.6 % decline in AOM cases was documented.^7^ The present findings align with these reports, demonstrating a 25,5 % reduction in AM incidence (March 2020–December 2021) compared to the previous 22 months.

It is reasonable to attribute this sharp reduction to social distancing measures, daycare and school closures, improved hygiene practices, and regular mask use in public spaces.

Recent studies from different countries indicate a post-pandemic resurgence of pediatric infectious diseases, often surpassing pre-pandemic levels. This trend applies to both viral and bacterial infections. In some locations, it has included an increase in cases of invasive streptococcal infections, as reported by the UK Health Security Agency.^14^ In France, after a decline in cases in 2020, health services reported a resurgence in bronchiolitis, gastroenteritis, and otitis, exceeding pre-pandemic figs.^15^ Similarly, a multicenter study in the United States documented a post-pandemic rise in acute pediatric rhinosinusitis.^16^ A German study further demonstrated that, in addition to an increase in upper respiratory tract infections, there was a rise in complications, surgical interventions, and the need for imaging exams (such as CT and MRI) compared to pre-2020 levels.^17^

Following this trend, the authors observed in this service a 25.5 % reduction in AM incidence during the pandemic (P2) compared to the pre-pandemic period (P1). However, this reduction was not statistically significant (*p* = 0.8027), likely due to the limited sample size. Conversely, there was a statistically significant 103.3 % increase in AM cases post-pandemic (P3) compared to baseline (P1) (*p* = 0.0322). Other factors, such as age, sex, complication occurrence, case severity, need for surgical intervention, antibiotic therapy duration, and hospitalization period, were not affected by the COVID-19 pandemic.

Some authors propose the concept of an “immunity gap” to explain this resurgence.^18^, ^19^, ^20^ This theory suggests that a prolonged absence or reduction of exposure to pathogens weakened immune system stimulation and reduced herd immunity. This could even be reflected in reduced cross-placental transfer of maternal antibodies to the fetus. This combination could lead to simultaneous susceptibility in a large population segment, potentially triggering disease outbreaks and straining healthcare systems.

Changes in seasonality patterns of endemic respiratory viruses have been documented, including respiratory syncytial virus, influenza A, and influenza B, which previously followed predictable cycles. Currently, their regional variability and unpredictable circulation periods pose challenges for developing effective public health response strategies.^19^^,^^21^^,^^22^

### Limitations and future challenges

This study has some limitations that should be considered in the interpretation of the findings. First, its retrospective design makes it susceptible to systematic biases, particularly selection and measurement bias. Additionally, as a single-center study, the findings may not be generalizable to the broader Brazilian population, given the country’s geographic, climatic, and socioeconomic diversity.

An important limitation of this study is the small sample size, which may have reduced the statistical power of these analyses. With only 39 cases included, the study is more susceptible to type II errors, especially in comparisons where clinically relevant differences were observed but did not reach statistical significance. This limitation should be considered when interpreting non-significant findings, as they may not truly reflect the absence of an effect.

Although long-term surgical outcomes were not assessed, the absence of reinterventions during hospitalization suggests immediate procedural success. Future studies should focus on long-term follow-up to better define functional and clinical outcomes. The surgical intervention rate in the studied cohort was 87.2 %, which may exceed rates reported in some international studies.^23^^,^^24^ This discrepancy may reflect the severity of cases treated in the tertiary care facility, the presence of complications at presentation, and specific institutional practices. Multicenter studies are needed to establish more precise benchmarks and further explore the variability in treatment approaches for pediatric acute mastoiditis.

Furthermore, although respiratory infections and AM are known to follow seasonal patterns, the authors did not analyze incidence trends on a month-by-month basis—an approach that might have added valuable insights, especially considering the distinct climate variability in southern Brazil. The authors also lacked data on personal or family history of COVID-19 prior to AOM/AM episodes, which could have contributed to understanding the immunological implications of prior infection.

One of the legacies of COVID-19 is the emergence of a broad field of unexplored knowledge that could enhance understanding of the endemic mechanisms of various pathogens and how individual and collective behavior can influence – both positively and negatively – the spread and virulence of these agents. Ongoing discussions on the multifaceted aspects of the pandemic can help strengthen medical, scientific, and policy frameworks to better prepare for future public health crises.

The COVID-19 pandemic and the behavioral changes it brought had a significant impact on the incidence of AM in the pediatric population treated at a tertiary referral hospital in southern Brazil. Following a decline in cases during the period of social distancing, a notable increase in incidence was observed compared to the pre-pandemic period. However, no significant changes were observed in the incidence of complications, disease severity, patient age, length of hospital stay, or duration of antibiotic therapy across the pre-pandemic, pandemic, and post-pandemic periods. Similar multicenter studies conducted in other regions of Brazil would be valuable to confirm this trend and contribute to the discussion on the immunological impacts of the pandemic and its long-term effects on pediatric public health in the country.

## Conflicts of interest

The authors declare that they have no conflicts of interest relevant to this study.

## Appendix Supplementary materials

Image, application 1

## Footnotes

Supplementary material associated with this article can be found in the online version at doi:10.1016/j.jped.2025.05.003.
