# Supplementary material for: Impact of the COVID-19 pandemic on the incidence of acute mastoiditis in a tertiary reference children’s hospital in Brazil
Source: J Pediatr (Rio J). 2025 Jun 28;101(5):101415. doi: 10.1016/j.jped.2025.05.003 (PMC12495564; doi:10.1016/j.jped.2025.05.003)
Supplement: Supplementary file 1 [file mmc1.docx]

**JPED-D-24-00305**

**SUPPLEMENTARY DATA**

| **SUPPLEMENTARY TABLE 1** | | |
| --- | --- | --- |
| **Quantitative comparison of patient age, the total length of stay, and total duration of antibiotic therapy in the pre-pandemic (P1) vs pandemic (P2), and pre-pandemic (P1) vs post-pandemic periods (P3) (Mann-Whitney test).** | | |
|  | **P1 vs P2 (p-value)** | **P1 vs P3 (p-value)** |
| Age (months) | 0.6993 | 0.4940 |
| Total duration of antibiotic therapy (days) | 0.1244 | 0.9533 |
| Total length of hospital stay (days) | 0.8935 | 0.3261 |

| SUPPLEMENTARY FIGURE 1 |
| --- |
| a) Incidence of acute mastoiditis per 100,000 pediatric hospitalizations across the pre-pandemic (P1), pandemic (P2), and post-pandemic (P3) periods.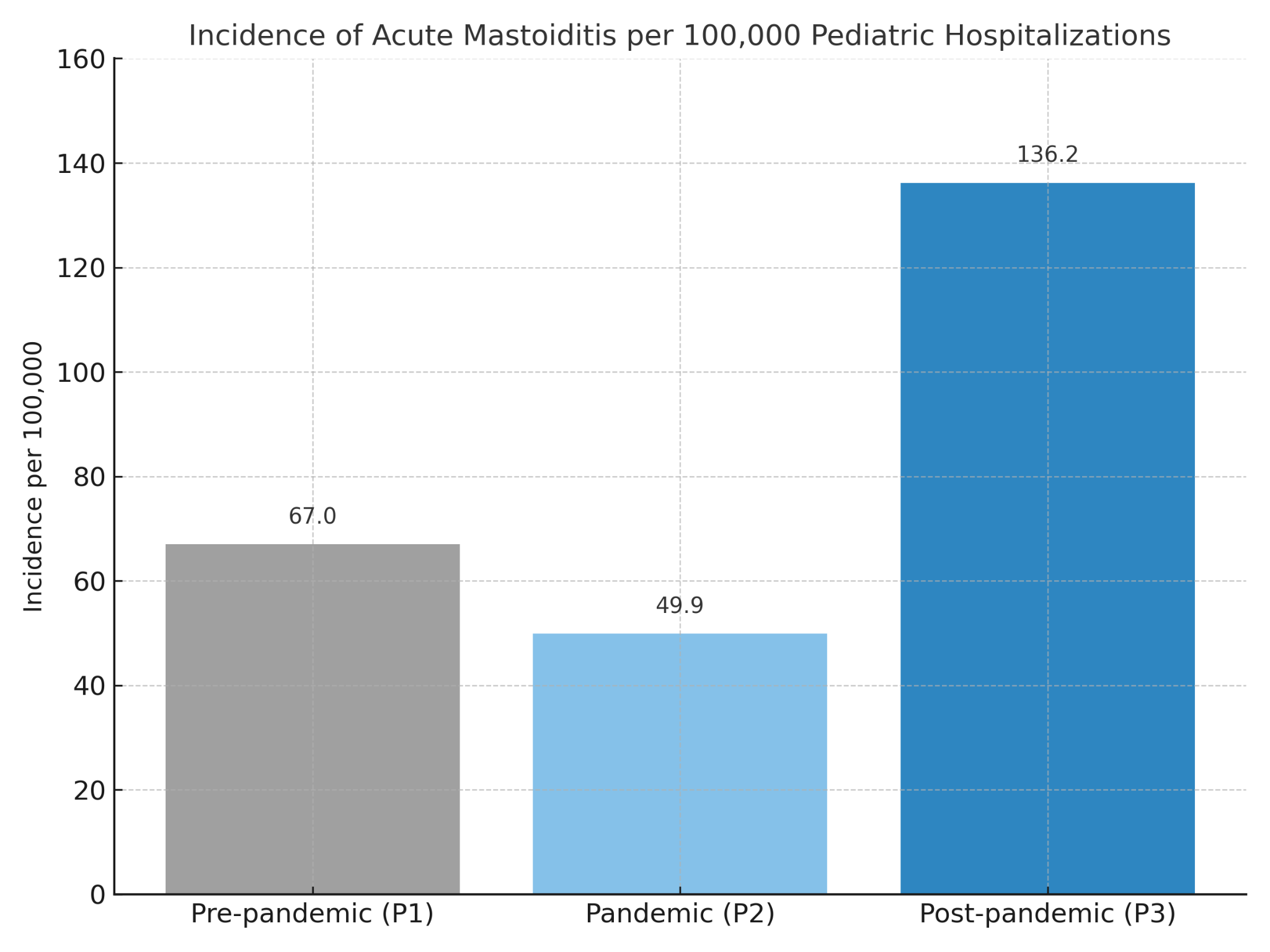 |
| b) Monthly average number of acute mastoiditis cases across the same periods.  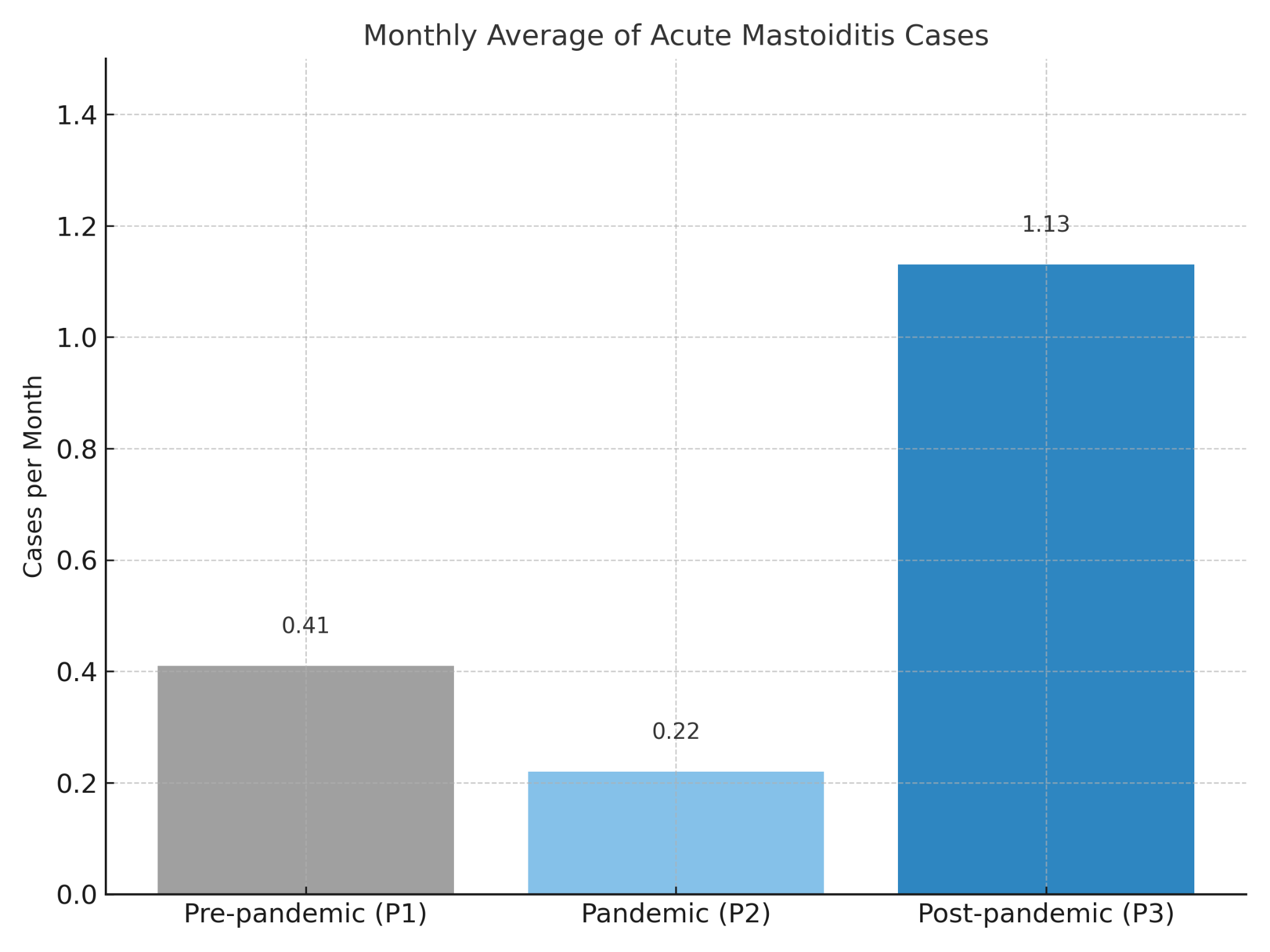 |
